# GenDrux: A biomedical literature search system to identify gene expression-based drug sensitivity in breast cancer

**DOI:** 10.1186/1472-6947-11-28

**Published:** 2011-05-05

**Authors:** Chiquito Crasto, Dajie Luo, Feliciano Yu, Andres Forero, Dongquan Chen

**Affiliations:** 1 Division of Research, Department of Genetics, Univ. of Alabama at Birmingham (UAB), USA; 2 Dept of Statistics, West Virginia University (WVU), USA; 3 Dept of Pediatrics, Children's Hospital, UAB, USA; 4 Comprehensive Cancer Center, UAB, USA; 5 Division of Preventive Medicine (DOPM), UAB, USA; 6 Clinical and Translational Science Institute, WVU, USA

## Abstract

**Background:**

This paper describes the development of a web-based tool, GenDrux, which extracts and presents (over the Internet) information related to the disease-gene-drug nexus. This information is archived from the relevant biomedical literature using automated methods. GenDrux is designed to alleviate the difficulties of manually processing the vast biomedical literature to identify disease-gene-drug relationships. GenDrux will evolve with the literature without additional algorithmic modifications.

**Results:**

GenDrux, a pilot system, is developed in the domain of breast cancer and can be accessed at http://www.microarray.uab.edu/drug_gene.pl. GenDrux can be queried based on drug, gene and/or disease name. From over 8,000 relevant abstracts from the biomedical literature related to breast cancer, we have archived a corpus of more than 4,000 articles that depict gene expression-drug activity relationships for breast cancer and related cancers. The archiving process has been automated.

**Conclusions:**

The successful development, implementation, and evaluation of this and similar systems when created may provide clinicians with a tool for literature management, clinical decision making, thus setting the platform for personalized therapy in the future.

## Background

### Cancer chemotherapy, gene expression, and drug sensitivities

Typically, diagnoses for most diseases often result from recognizing disease phenotypes, though these phenotypes might arise from a range of gene expression profiles [1]. Treatment modalities for cancer such as chemotherapy can be personalized to the gene expression profile of a patient, if sensitivity of a pharmaceutical product can be associated specific genes, treating the underlying cause that stems from a patient's specific genotype. This is one of the means by which the eventual goals of personalized medicine, recognition of disease-gene relationships, gene-drug relationships [2], and the disease-gene-drug nexus [3], may be achieved. Table 1 represents salient examples of an increasing number of studies that have shown that recognizing gene-drug and disease-gene relationships can be potentially useful to clinicians. A search in PubMed using the phrase "breast cancer and drug" results in nearly 57,000 abstracts returned, of which nearly 10% are between 2009 and 2010. This is indicative of a greater emphasis on increasing recent work on gene expression contributions to disease within the research and clinical communities. References to new discoveries of these relationships are often available only in the non-annotated, free text of the biomedical literature. Manual identification of this information is challenging. It involves surveying thousands of articles to identify very specific relationships. The resulting information has to be processed, archived and efficaciously retrieved so that it is available to clinicians and researchers.

**Table 1:** Disease-Gene Expression-Drug Sensitivity Relationships in Breast Cancer

	Disease	Gene	Drug	Sensitivity*	**Ref**.
1	Breast cancer	estrogen-induced genes	Estrogen	increase	[8]

2	Breast cancer	Her-2/ErbB2	Tamoxifen	decrease	[9]

3	NSCLC, gastric	ERCC1, BRCA1	Cisplatin, docetaxel	Decrease	

4	Breast cancer	estrogen receptor, progesterone receptor, HER2, Ki-67, p53, and bcl-2	Doxorubicin, docetaxel, tamoxifen	decrease (estrogen R, Kip67), increase (Bcl2)	[10,11]

5	Breast cancer	Glutathione peroxidase	DHA, doxorubicin	increase	[12]

6	Breast cancer	Aromatase Inhibitor, pS2	Letrozole	increase	[13]

**Decrease **suggests that increased expression of the mentioned gene decreases the drug sensitivity, or makes the drug more resistant.

Several drug-gene-disease relationship tools are currently available. PharmGKB[4,5] is a resource that provides information related to genotypes and phenotypes in disease, disease related pathways as well as information related to pharmaceutical products. Lingpipe (http://alias-i.com/lingpipe/index.html) is a resource that allows open source access to natural language processing algorithms, one of which is named entity recognition (NER). Although it uses LingMed that enables extraction of Medline literature similarly as the GenDrux, described here, its target audience is not clinicians. Additionally, BANNER and ABNER are systems that use NER to identify gene and protein names to identify protein-protein interactions. BANNER[6] has to be downloaded and installed. ABNER [7] is an open source tool for automatically tagging genes, proteins, and other entity names in text.

These sophisticated systems are subject to issues of precision and recall if the entity is incorrectly identified. Systems such as PharmGKB are manually curated and its sources are myriad. Such resources are also comprehensive and not likely to be immediately useful to specialized clinicians. Additionally, while these systems are free and open source, they have to be downloaded and installed on the users' computer. A browser based search and retrieval system that is platform independent and requires only a browser would likely be preferable.

### Maintenance and update

In GenDrux the drug names as well as the gene names are extracted together with article PubMed IDs, article titles and abstracts, from resources that are validated repositories of this information and therefore not subject to uncertainties or ambiguities. GenDrux, in its current state, and when fully evolved, depending on how quickly gene expression data and pharmaceutical product development keep up, will ensure accuracy of information retrieved. GenDrux, is currently applied to breast cancer, but is easily extensible to other diseases. The tool can be accessed over the Internet through a Web browser. The system is queried by means of a simple form. GenDrux has been tested for all the available browsers in different computer platforms. This system lends itself well to use by physicians and researchers.

## Implementation

The objective was to create a Web-based literature archiving and retrieval system that could be used by clinicians and researchers to search for abstracts that depicted research that identified drug-gene relationships. Our basic methodology consists of two steps; 1) automated creation of the archive of the relevant biomedical literature; and 2) creating the web-based query tool for retrieval of information in response to users' queries. There is no client software to install.

### Archiving the biomedical literature

To archive relevant literature in the domain of breast cancer, we leveraged two tools (E-Search and E-Fetch), from the E-utils suite, developed at the National Library of Medicine (NLM). E-utility allows the extraction of abstracts from PubMed in an automated fashion. We developed a "wrapper"--a PERL (Practical Extraction and Report Language) program that incorporated e-utils' features.

### E-Search

Here parameters that are used to design a PubMed search can be embedded in a universal resource locator (URL). These parameters (of many possible parameters) include search phrases (depicting the domain of research, e.g., a disease, in this case "breast cancer"), a range of dates, the journal, one or more authors, and the maximum number of hits (in our case, 500, which is the maximum allowed) returned. For example, our e-search URL was as follows: http://eutils.ncbi.nlm.nih.gov/entrez/eutils/esearch.fcgi?db=pubmed&term="breast+cancer+genes+drug"&minddate=0&maxdate=$date[1]&retmax=500.

Since our aim is to identify pharmaceutical products that might be related to specific genes implicated in breast cancer, our search phrases consisted of "breast+cancer+gene+drug." The "+" sign is used as an "AND" during the search. In order to ensure that we could extract as many abstracts as possible, our algorithm looped over 500 abstracts retrieved per search for specific time periods starting from 1975 to the present. In determining how many results would adequately represent the entire corpus of relevant literature, from 1975 to 1985 we searched twice a year, up to 1995, four times a year and from then on, monthly. E-utils results are retrieved as XML (eXtensible Markup Language) files. We developed a PERL based XML parser that processed the E-Search results file. This results file consists of a list of unique (and dynamically created) identifiers. Each identifier represents a publication stored in PubMed and can be "fetched" as an abstract.

### E-Fetch

The results from E-search were further processed to automatically download the abstracts. For each identifier we developed another PERL "wrapper" that incorporated the e-fetch URL. In the following example of the e-fetch URL: "http://www.ncbi.nlm.nih.gov/entrez/eutils/efetch.fcgi?db=pubmed&id="Unique_identifier"&rettype=abstract&retmode=XML", the program fetches information about a publication for a "unique_identifier" from the E-Search result. This information includes all the information about a publication that a user would access in PubMed. We created another PERL-based XML parser to extract from the downloaded file: the title of the paper, the abstract, the first (lead) author name and the last (presumptive ranking) author, and the journal name. This information was added to the archive file in the format: ***| PubMed links | gene name| drug name | authors | date of publication | journal name | title of publication |***. This archive file serves as the candidate article database and a screening tool that is accessed by the GenDrux web-retrieval system. This archive can be modified for other diseases by changing the original search phrase ("breast+cancer+gene+drug") in E-search.

### Filtering the archive file for relevance

The archive file containing the abstracts was further processed so each abstract and title contained a valid gene and drug name associated with breast cancer. We filtered the archived abstracts and titles only to those that were listed in the gene annotation files (from human genome array from Affymetrix, e.g.), NCBI, Ensemble, and GEO (Gene Expression Omnibus). In addition, we used a compendium of gene synonyms and aliases to improve recall. In the text of the titles and abstracts, gene names, gene symbols, aliases, Unigene IDs were all treated as equal. That is, if the file contained an article to a gene (e.g., Unigene ID), that article was mapped for that record as being associated with that gene.

The archive file was further filtered to restrict the abstracts that contained gene information in the domain of breast cancer, which also include names of drug names. For this, we enlisted a comprehensive list of names of pharmaceutical products using a database from the Food and Drug Administration (FDA), the National Cancer Institute (NCI) and the International Classification of Diseases (ICD) web resources. The final archive file contained articles that only referenced specific genes (by their names and aliases) and drugs.

### Interface and system design

**Figure 1:**
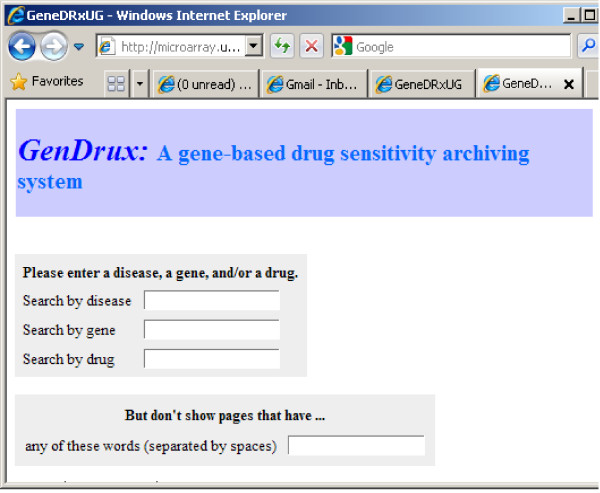
**The Web interface for the GenDrux system**.

The archive file, created as described in the previous section, was stored on a Windows-based Internet Information Server (IIS) server. It is a single flat file that contains information that will constitute a user's query. The Web interface (Figure 1) was created using a PERL program that invoked the CGI.pm module. The module helps to create web-based query-able interface. The GenDrux system design is simple. It contains two files: the Web-retrieval program and the archive file. The system allows users to choose and exclude keywords to further filter the results. The results are available instantly and dynamically in the tabulated format.

## Results and Discussion

In the creation of the archive file, approximately 8,000 abstracts were downloaded and then reduced to approximately 4,000 records, using gene name (against 24,000 records) and drug name (~3,050 records) filters. The number of abstracts in the archival file was reduced to approximate 4,000. Each record corresponds to a unique publication. This is not far from the number that a user browsing through the PubMed web pages might find, where a "breast cancer AND gene AND drug" search retrieves a little over 9000 abstracts, a little over a thousand relevant articles having been added to PubMed since the archive was created (this will be updated at regular interval). Our decision to extract twice in a year from 1975 to 1985, four times a year between 1986 to 1995 and every month from then on appears justified.

### Web-based query and visualization procedure

**Figure 2:**
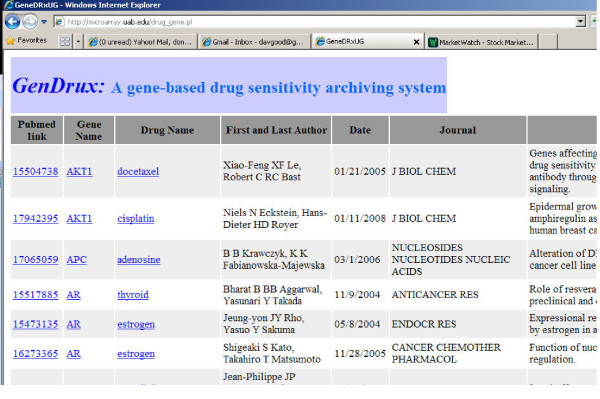
**The searching results by a disease**.

The GenDrux Web portal can be found at http://microarray.uab.edu/drug_gene.pl. The front page of GenDrux is shown in Figure 1. A user can search the downloaded literature using one or more parameters: disease, drug name, and gene name. A negative control feature is also available, which allows a user to exclude abstracts that contain specific keywords or phrases. The results include the title, the abstract, the gene name, drug name, the date of publication, the name of the first and ranking author and the journal name, as shown in Figure 2. Each result also includes a link to the PubMed abstract, as well as link to drug.com and (Gene Ontology) GO website. For academic users or those who subscribe to the journal, this link will also lead the user (through PubMed) to the full-text of the article. The system identifies articles that contain references to genes and drugs for specific cancer. Given the nature of information retrieval from free-text (this is discussed in detail later), the system relieves the burdens of processing large amounts of text. We leave it to the clinician-user to determine the specific relationship or whether the relationship merits a diagnosis or a prescription.

A user on entering the terms such as "BRCA1" or "BRCA2" gene names will obtain results, with those terms highlighted in the title in GenDrux's results page. The same applies to the name of a drug. The PERL program looks for exact matches before returning results. The decision to use exact as opposed to partial matches is because the names of genes and drugs are tokens that are precisely named, as opposed to tokens such as parts of speech, etc. Discussed here are two cases where the retrieval methodology can be used to extract useful information and draw conclusions.

### User case 1 (Search for a drug name)

Consider a search on the drug "etoposide". The GenDrux system returns 14 results. Etoposide is a chemotherapeutic drug that is responsible for DNA repair and has been useful in different forms of cancer. Each of the results corresponds to a different gene, but the same drug. This is an interesting observation: in addition to the knowledge desired, i.e., the publications associated for this drug, etoposide is shown to be implicated in treatments associated with several genes. This is a possible comment on the 'one size fits all' approach to chemotherapy: different gene expression profiles might give rise to the same phenotype, and the chemotherapy modality that uses a specific drug (e.g., etoposide) might not be beneficial to all.

### User Case 2 (Search for disease names)

The archive file was created following a search for articles related to breast cancer. It was further filtered to abstracts containing the genes and drug names that have been validated and established by several entities. A search in GenDrux for "colon cancer" produces 23 results and search for "leukemia" produces 27 results. On the other hand, a search for "liver cancer" does not produce any results. Physicians and other users can this use GenDrux to make assessments related to possible correlations between the two cancers. At least superficially, there seems to be no correlations between breast and liver cancers, but there appears to be some between the two cancers.

The primary purpose of GenDrux is to retrieve relevant gene-drug relationships for specific diseases like breast cancer, out of the vast biomedical literature. The tool helps focus the users' query. It is up to the users, a physician, e.g., to identify the parameters for correlations between two types of cancers or the relationship between a gene that is over or under expressed and a drug that is used in the treatment of the cancer. A confirmed laboratory-result for a gene may increase confidence for a relevant prescription decision. These relationships involve expert understanding about very specific aspects of a domain and cannot be discerned from this tool. GenDrux, however, retrieves information in an automated fashion. This saves times and offers a pool of candidate relationships for users to study and archive.

This system, developed as a pilot system using breast cancer as a test-bed, searches, documents, archives, and updates the gene-drug relations automatically. GenDrux relies on a NER scheme, but does not process the natural language of the biomedical text. It does not make artificial intelligence-type decisions as to the relationship between a gene profile and its sensitivity to a drug. When PubMed identifies relevance to a keyword or concept using MeSH (Medical subject heading) term, its curators do so manually. A more careful perusal of an article will reveal that the gene name and the drug reference are not in the same sentence or in the same paragraph. The goal of the system, one anticipates, is more to prevent using medication that implies resistance when certain gene expression profiles exist, rather than to suggest a medication that has been implicated sensitive when another gene expression profile exists. GenDrux, as currently developed, relies on the physician and/or researcher to draw conclusions from the text of the literature that identifies a gene and a drug, as to the impact of the drug on the gene, and vice versa. The system, when fully optimized, may incorporate high throughput gene expression profiles as well as new drug-related research information to impact the underlying cause for drug sensitivities, and may ultimately provide physicians with guidelines for prescriptions.

The system provides Web access in a secure way and is cost effective due to its automated search and retrieval mechanism. The web page contains a link to a survey and a link to the email address of the primary developer. The latter is to address changes and improvements based on user-requests. Users can seek help and provide feedbacks in using the system as well as interpreting the results.

One other advantage of this system is that it does not need downloading and installing software. The system is platform independence and is scalable. Changing of search terms from "breast cancer" into other diseases, during archive file creation, will make the system useful in that disease domain. The automated download process with the e-search and e-fetch URL embedded in the Perl wrapper can be used similarly to download abstracts related to other diseases or research domains.

The system has its limitations as well. Currently, it is focused on breast cancer. Only the search keywords (disease name, drug name, etc) in the URL in e-search, however, need to be changed. Once the abstracts are downloaded, they can be processed in a text file or a table in a database. For this prototypical system, given the sources for gene and drug names, the results are universally deemed as reliable. Secondly, the system provides a tool of querying candidate gene-drug relations, which by no means indicate a direct clinical application. More validation and/or manual annotation and confirmation may be needed for clinical decision support in personalized breast cancer chemotherapy in the future.

## Conclusions

We developed and implemented a prototype system that searches and archives gene expression and drug sensitivity information. The system is designed to inform a physician such reported gene-drug relationships. The query results from the system provide physician linkages to the articles and help them determine their clinical implications. The prototype system that focused on breast cancer can be extended to other cancer s or non-cancer diseases.

## Availability and requirements

The source code for GenDrux is available for downloading for non-commercial purposes. Please contact authors (CC and DC).

• Project name: GenDrux

• Project home page: http://www.microarray.uab.edu/drug_gene.pl.

• Operating system(s): Platform independent.

• Programming language: Perl.

• License: free for academics.

## List of abbreviations

MeSH: medical subject headings; XML: Extensible Markup Language; PERL: practical extraction and report language; IIS: Internet Information Server; NDC: National Drug Code; SNOMED: Systemized Nomenclature of Medicine.

## Competing interests

The authors declare that they have no competing interests.

## Authors' contributions

DC is principal investigator of the project. DC, CC, and FY designed the system. CC is the primary developer for the archiving and web-program. All algorithms are developed using the PERL programming and scripting language. DC implemented IIS and Perl on server. FY and AF evaluated the system. All authors contributed to overall design, development, and implementation of the system. All authors read and approved the final manuscript.

## Pre-publication history

The pre-publication history for this paper can be accessed here:

http://www.biomedcentral.com/1472-6947/11/28/prepub
